# Efficacy of Triple Antibiotic Paste and Bromelain Paste As Intracanal Medicament Against Enterococcus faecalis: An In-Vivo Study

**DOI:** 10.7759/cureus.48098

**Published:** 2023-11-01

**Authors:** Pradnya Nikhade, Paridhi Agrawal, Joyeeta Mahapatra, Tejas Suryawanshi, Jay Bhopatkar, Laxmikant Umate

**Affiliations:** 1 Department of Conservative Dentistry and Endodontics, Sharad Pawar Dental College and Hospital, Datta Meghe Institute of Higher Education and Research (DMIHER), Wardha, IND; 2 Department of Research and Development, Datta Meghe Institute of Higher Education and Research (DMIHER), Wardha, IND

**Keywords:** antimicrobial, intracanal medicament, enterococcus faecalis, triple antibiotic paste, bromelain

## Abstract

Introduction

Microorganisms are pivotal contributors to pulp and periapical pathology, often culminating in endodontic treatment failures. The successful outcome of such treatment hinges on the effective eradication of microbial flora through various interventions, including intracanal medicaments (ICMs). However, *Enterococcus faecalis* (*E. faecalis*), a highly adaptive Gram-positive bacterium, exhibits remarkable resilience and can persist even after rigorous disinfection efforts. This study explores the efficacy of two ICMs, calcium hydroxide-based triple antibiotic paste (TAP) and a novel alternative containing bromelain, derived from pineapples, in combating *E. faecalis* infections in vivo.

Methodology

This in-vivo study was conducted at Sharad Pawar Dental College and Hospital, Wardha, India, and ethical approval was obtained from the institutional ethical committee. The sample size was calculated using the OpenEpi program (version 3.04.04, Open Source Epidemiologic Statistics for Public Health, www.OpenEpi.com), resulting in 15 participants per group. Inclusion criteria encompassed mandibular premolars with carious involvement but no prior restorations, diagnosed with necrotic or infected pulp, and no significant medical history. Exclusion criteria included systemic conditions, pregnancy, retreatment cases, recent antibiotic therapy, calcified canals, and other contraindications. After proper patient consent, access opening and chemomechanical preparation were performed, and root canals (RCs) were randomly divided into two groups: TAP and bromelain paste (BP). TAP was prepared using ciprofloxacin, metronidazole, and minocycline, while BP comprised bromelain powder mixed with saline. Both groups received intracanal medicaments, followed by temporary sealing. Microbiological samples were collected before and after treatment for analysis.

Results

*E. faecalis* count (CFU/mL) before instrumentation for the TAP group was (1.94 x 10^5^ $\pm$ 7.45 x 10^3^) and for BP group was (1.97 x 10^5^ $\pm$ 7.70 x 10^3^) with p-value 0.26 $\geq$ p=0.05, so no significant difference was found between them. *E. faecalis* count (CFU/mL) after instrumentation for the TAP group was (7.70 x 10^3^ $\pm$ 9.11 x 10^2^) and for the BP group was (7.26 x 10^3^ $\pm$ 8.43 x 10^2^) with p-value 0.18 $\geq$ p=0.05, so no significant difference was found between them. However, the *E. faecalis* count obtained after seven days of intracanal medicament for the TAP group was (3.63 x 10^1^ $\pm$ 5.60) and for the BP group was (3.13 x 10^1^ $\pm$ 4.55) with p-value 0.012 < p=0.05, so a significant difference was found between them. This means that when compared with the TAP group, there was a greater amount of reduction in *E. faecalis* count (CFU/mL) for the BP group seven days after the placement of intracanal medicament, and this reduction was statistically significant.

Conclusion

This in-vivo study highlights the potential of BP as a more effective intracanal medicament against *E. faecalis* when compared to the conventional TAP. Bromelain's selectivity for Gram-positive bacteria and its diverse therapeutic properties make it a promising natural alternative for endodontic treatment. Further research is warranted to optimize bromelain's clinical application and assess its potential to enhance endodontic outcomes, potentially revolutionizing the field of endodontics.

## Introduction

Microorganisms play a vital character in the development of pulp and periapical pathology and can lead to failures in endodontic treatment. The effectiveness of such a treatment hinges on effectively eradicating microbial flora through various irrigation methods, activation techniques, and careful application of intracanal medicaments (ICMs) [1,2]. Nevertheless, specific microbes, such as *Enterococcus faecalis* (*E. faecalis*), may persist even after careful disinfection, contributing to endodontic failures [3,4]. *E. faecalis*, a Gram-positive facultative anaerobe, exhibits adaptability to changing environments and has the capacity to firmly breach dentinal tubules, making it challenging to eliminate [5,6].

In the pursuit of limiting microbial residue, various ICMs, such as calcium hydroxide (CaOH) [7], formocresol, chlorhexidine [8], glutaraldehyde, and propolis [9], have been investigated. Ideally, these ICMs should possess continuous and extended antimicrobial effects, solution stability, biocompatibility, and resistance to staining. However, no perfect ICM has been identified thus far [10]. CaOH, the most commonly used ICM, has been shown to hinder the root canal (RC) system's strength over time [2,10]. Conversely, triple antibiotic paste (TAP), which has demonstrated efficacy, can lead to microbial resistance and tooth discoloration [11]. Given the need to balance safety and effectiveness issues with synthetic agents, herbal alternatives may offer advantages.

Numerous herbal agents, including propolis, triphala, *Camellia sinensis* (tea), neem, guava, orange oil, and turmeric, known for their potent antimicrobial properties, have been employed for canal disinfection [12]. Although there are few reports of adverse effects, such as allergic contact dermatitis, allergic conjunctivitis, and digestive tract irritation in the literature [13], these natural products are generally considered safe. In addition, bromelain, an underexplored agent, boasts proteolytic, anti-inflammatory, antibacterial, fibrinolytic, antithrombotic, and antifungal properties [14].

Bromelain, produced from the pineapple fruit and stem (*Ananas comosus*) and part of the Bromeliaceae family [15], has diverse applications, including the treatment of allergies, inflammation, burns, blood coagulation, sinus congestion, diarrhea, osteoarthritis, cardiovascular diseases, and cancer [16]. It is also recognized for enhancing the uptake of antibiotics, resulting in improved distribution of the medication in body tissues and consequently reducing the potential adverse effects linked to its toxicity [17]. Its rich content of flavonoids and protease contributes to its antibacterial properties. Research has explored its effectiveness against periodontal microbes [18] and in teeth bleaching [19]. Given its versatility and exceptional properties, bromelain cannot be ignored as a potential option in the field of endodontics.

This in-vivo study aims to compare the antimicrobial activity of TAP and TAP with bromelain, a naturally available potent proteolytic, anti-inflammatory, and antibacterial extract derived from pineapples, against* E. faecalis* bacteria as an ICM.

## Materials and methods

This research study obtained ethical approval from Datta Meghe Institue of Medical Sciences Institutional Ethics Committee (approval number DMIMS(DU)/IEC/2022/75). The study was carried out within the Department of Conservative Dentistry and Endodontics at Sharad Pawar Dental College and Hospital, Wardha, India. The OpenEpi program (version 3.04.04, Open Source Epidemiologic Statistics for Public Health, www.OpenEpi.com) was used to calculate the sample size, following a methodology outlined in a study [20], with a 95% confidence interval (CI) and 80% statistical power, and the sample size per group was calculated to be 15 individuals. All participants involved in the study were duly informed about the experiment, and their written informed consent was obtained.

Inclusion and exclusion criteria

In this study, the inclusion criteria encompassed patients with mandibular premolars that exhibited carious involvement but had no prior history of restorations. These teeth were diagnosed clinically and radiographically as having necrotic or infected pulp. Patients without any notable medical history were considered for inclusion.

Conversely, patients with known systemic conditions; pregnant women; cases requiring retreatment; individuals on antibiotic therapy within the three months leading up to the study; teeth with calcified canals, immature apex, periapical lesions, sinus openings, and any indications of external or internal resorption; and teeth exhibiting grade III mobility were excluded from participation in the study.

Methodology

Following local anesthesia and the establishment of rubber dam isolation, the access cavity was meticulously prepared, allowing access to the pulp chamber. To facilitate the insertion of paper points into the canal, the canal orifices were enlarged using hand SX files (Dentsply, Sirona). Subsequently, the first microbiological sample (S1) was collected by gently inserting a size #30 (6%) sterile absorbent paper point into the canal, leaving it in place for approximately one minute, and then transferring it into a test tube containing brain heart infusion (BHI) broth.

The second microbiological sample (S2) was collected after performing chemomechanical preparation using rotary NiTi files (EdgeEndo, USA) along with a combination of 2.5% sodium hypochlorite and 0.9% normal saline. Following this step, the RCs were randomly divided into two groups, each comprising 15 samples. Intracanal medicaments were placed as follows:

Group 1

For the preparation of the TAP, three commercially available drugs, namely, ciprofloxacin (Cifran 500 mg, Ranbaxy Laboratories Limited, India), metronidazole (Metrogyl 400 mg, JB Chemicals and Pharmaceuticals Limited, India), and minocycline (Minoz 100 mg, Ranbaxy Laboratories Limited, India), were utilized. The enteric coating of these drugs was removed, and each drug was pulverized under aseptic conditions. These powdered drugs were then mixed in a 1:1:1 weight-to-volume ratio to create the TAP. This paste was further mixed with 0.3 mL of normal saline (0.9%) and placed in the canal.

Group 2

The BP was prepared using bromelain powder (Brisk Bioscience, Surat, Gujarat, India) with an enzymatic activity of 2400 gelatin digestion units per gram. This powder was mixed with saline in a 1:1 proportion, meaning that 1 gram of powder was mixed with 1 mL of normal saline (0.9%), and the resulting paste was placed in the canal.

To ensure a secure seal, a double temporary seal was created using NeoTemp (NeoEndo, India) and glass ionomer cement (Ketac-Fil, 3M ESPE, USA), which remained in place for seven days. After the seven-day period, the temporary dressing was removed, and thorough irrigation with normal saline was performed to completely remove the intracanal medicament. Following this, the third sample (S3) was collected.

All test tubes containing microbiological samples were securely sealed, appropriately labeled, and sent for microbiological analysis. These test tubes underwent a pre-incubation step for 30 minutes at 37°C and were then vigorously shaken for 60 seconds in a vertex mixer. Serial 10-fold dilutions were carried out up to a 1:10^6^ dilution factor in 1% sterile saline. From these serial dilutions, 0.1 mL was aseptically transferred onto blood agar and MacConkey agar plates. The plates were subsequently incubated for 24 hours, after which all plates were assessed for the presence of colony-forming units (CFUs) per mL. The identification of the organisms (*E. faecalis*) was achieved through Gram staining, catalase production, and colony morphology assessment on blood agar using bile esculin agar.

Statistical analysis

Statistical analysis was done with IBM SPSS Statistics for Windows, version 21 (released 2012; IBM Corp., Armonk, New York, United States) at 95% CI and 80% power to the study. The outcome variables (CFU/mL) count before instrumentation, after instrumentation, and after placement of intracanal medicament were tested for normality using Kolmogorov-Smirnov test and Shapiro-Wilk test, and it followed a normal distribution as the P-value tested showed non-significant results (P≥0.05). Thus, parametric test one-way analysis of variance (ANOVA) was used for the evaluation of *E.faecalis *count (CFU/mL). Statistical significance was calculated at ≤0.05.

## Results

**Figure 1:**
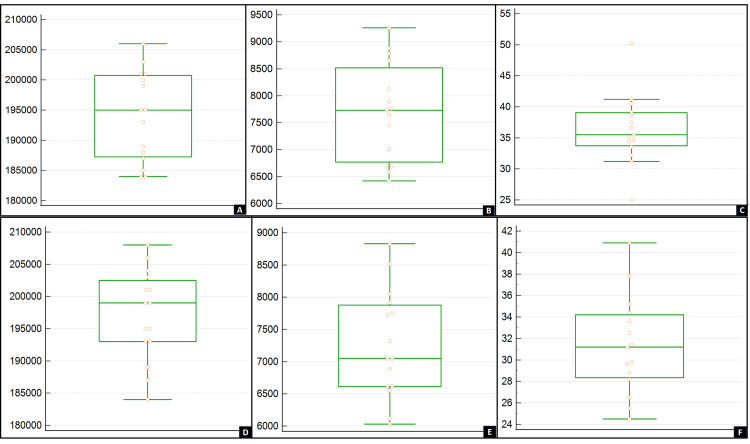
Box-whisker plot of the results of the triple antibiotic paste group and bromelain group A: *Enterococcus faecalis* count (CFU/mL) before instrumentation (S1) for the TAP group. B: *E. faecalis* count (CFU/mL) after instrumentation (S2) for the TAP group. C: *E. faecalis* count (CFU/mL) after seven days of intracanal medicament (S3) for the TAP group. D: *E. faecalis* count (CFU/mL) before instrumentation (S1) for the BP group. E: *E. faecalis* count (CFU/mL) after instrumentation (S2) for the BP group. F: *E. faecalis* count (CFU/mL) after seven days of intracanal medicament (S3) for the BP group. TAP: triple antibiotic paste; BP: bromelain paste

Figure 1 depicts the comparative evaluation of *E. faecalis* counts obtained from the RCs before instrumentation (S1), after instrumentation (S2), and after seven days of intracanal medication (S3) for both groups (TAP and BP) to find significant results using the one-way ANOVA test.

The *E. faecalis* count (CFU/mL) before instrumentation for the TAP group was (1.94 x 10^5^ $\pm$ 7.45 x 10^3^) and for the BP group was (1.97 x 10^5^ $\pm$ 7.70 x 10^3^) with p-value 0.26 $\geq$ p=0.05, so no significant difference was found between them. The *E. faecalis* count (CFU/mL) after instrumentation for the TAP group was (7.70 x 10^3^ $\pm$ 9.11 x 10^2^) and for the BP group was (7.26 x 10^3^ $\pm$ 8.43 x 10^2^) with p-value 0.18 $\geq$ p=0.05, so no significant difference was found between them. However, the *E. faecalis* count obtained after seven days of intracanal medicament for the TAP group was (3.63 x 10^1^ $\pm$ 5.60) and for the BP group was (3.13 x 10^1^ $\pm$ 4.55) with p-value 0.012 < p=0.05, so a significant difference was found between them. Hence, compared with the TAP group, there was a greater amount of reduction in *E. faecalis *count (CFU/mL) for the BP group seven days after the placement of intracanal medicament, and this reduction was statistically significant.

## Discussion

The primary objective of endodontic treatment is the thorough elimination of microorganisms from the RC system. This goal is accomplished through an extensive chemo-mechanical preparation process, which is subsequently followed by the comprehensive three-dimensional sealing of the RC system. ICMs serve as supplementary measures to eradicate or diminish the microbial load within the RC system before the final sealing process. They target any residual microorganisms that may persist after the chemo-mechanical procedures, thus reducing inflammation and mitigating the risk of reinfection. Notably, *E. faecalis* is the predominant microorganism observed in cases of persistent or recurrent endodontic infections [1]. As the most resilient intracanal pathogen encountered in cases of unsuccessful RC treatments, *E. faecalis* stands as the benchmark for representing other potential microorganisms in these situations. Due to the pressing need for an effective therapeutic solution against *E. faecalis*, evaluating this specific microbe for its potential susceptibilities is of great importance [2]. Therefore, in this current study, *E. faecalis* was employed as the standard bacterium against which the experimental ICMs were evaluated.

TAP serves as a commonly employed intracanal medication in the field of regenerative endodontics. It comprises a combination of ciprofloxacin, metronidazole, and minocycline, which is a derivative of tetracycline. Extensive research has been conducted to assess the effectiveness of this blend against a variety of endodontic microorganisms, consistently demonstrating its advantageous properties [21]. Previous studies have stated that TAP effectively eliminates *E. faecalis* from the RC [22].

In the present study, the results indicated that bromelain was more effective than TAP (p=0.012). According to existing literature, bromelain has demonstrated greater effectiveness against Gram-positive bacteria compared to Gram-negative ones [15]. *E. faecalis*, classified as a Gram-positive bacterium, is notorious for its resistance to drugs, making it challenging to treat. Bromelain's selectivity toward Gram-positive bacteria presents an additional advantage for its potential efficiency against *E. faecalis*, complementing its other benefits [23]. In a prior clinical trial, oral bromelain was observed to reduce postoperative erythema, pain, and inflammation following third molar extraction [24]. The antibacterial properties of bromelain are attributed to its chemical compounds, including saponins, tannins, flavonoids, and various enzymes. Flavonoids, in particular, possess the inherent ability to form complex bonds with extracellular proteins through hydrogen bonding, consequently altering cell membrane permeability [25]. In the present study, it is worth noting that bromelain exhibited statistically significant antimicrobial effectiveness against *E. faecalis* when compared to TAP. It is important to mention that no prior studies have directly compared the antimicrobial efficacy of TAP with bromelain. Further clinical research involving varying concentrations, different modes of delivery, and different durations for which bromelain is placed as an intracanal medicament is warranted to confirm its effectiveness against the highly resistant *E. faecalis*. A shorter follow-up period, with no negative control, and a smaller sample size is a limitation of this study.

## Conclusions

This in-vivo study compared the effectiveness of TAP and BP as intracanal medicaments against *E. faecalis* in endodontic treatment. Our findings suggest that BP demonstrated superior antimicrobial efficacy when compared to TAP. This promising result highlights the potential of bromelain as an alternative intracanal medicament, offering a safe and effective solution in the ongoing quest to combat persistent endodontic microorganisms. Further research is essential to validate and optimize the use of bromelain in clinical practice, potentially improving the outcomes of endodontic treatments. Embracing natural alternatives, such as bromelain, could revolutionize the field of endodontics.
